# Prophylactic Anticoagulation in Nephrotic Syndrome

**DOI:** 10.34067/KID.0000000000000225

**Published:** 2023-10-26

**Authors:** Jonathon Gerstein, Kiran Battu, Heather N. Reich

**Affiliations:** 1 Department of Medicine, University of Toronto, Division of Nephrology, University Health Network, Toronto, Ontario, Canada; 2 Department of Pharmacy, University Health Network, Toronto, Ontario, Canada; 3 Division of Nephrology, University Health Network, Toronto, Ontario, Canada

**Keywords:** glomerulonephritis, membranous nephropathy, nephrotic syndrome, primary glomerulonephritis, thrombosis

Important questions remain about the benefits and approaches to prophylactic anticoagulation (PAC) in adults with nephrotic syndrome (NS). New data regarding the pharmacokinetics of direct factor Xa oral anticoagulants (DOACs) in NS merit a review of our knowledge in this realm in the context of current clinical guidelines and a need for trials to address gaps in knowledge.

## What is Known?

Hypercoagulability is a well-known complication of NS, conferring both increased venous and arterial thrombotic risk. The largest epidemiologic studies suggest that clinically apparent venous thromboembolic (VTE) events occur in up to 7.2% of adults with membranous nephropathy (MN), although the rate of subclinical VTE events is significantly higher when serial radiologic screening is applied.^1^ The risk of VTE events is over 10× higher in MN and 6× higher in FSGS compared with IgA nephropathy.^2^

A consistently identified risk factor for the development of VTE events across histologic subtypes of GN is the degree of hypoalbuminemia. The highest risk of events is observed in patients with MN with a serum albumin of <22 g/L.^3^ VTE events in these cohorts occurred^4^ primarily within 4.5 months of diagnosis (88%).

Hypoalbuminemia affects the pharmacokinetics of warfarin; patients with hypoalbuminemia treated with warfarin are at increased risk of supratherapeutic anticoagulation and major bleeding.^4^ Frequent blood monitoring may be required to minimize this risk, but this is cumbersome for patients and clinicians prompting a search for safer and more convenient anticoagulants.

It is now recognized that patients with MN and other forms of GN are also at increased risk of cardiovascular events, even in the absence of NS.^5^ Patients with biopsy-proven GN have a 2.5-fold risk of cardiovascular events compared with age-matched and sex-matched populations.

## Knowledge Gaps

The nature of the imbalance between thrombophilic and fibrinolytic factors leading to the thrombophilic state associated with NS is poorly understood. This contributes to a knowledge gap regarding the safest and most effective approaches to prevention. In the absence of randomized clinical trials, we are left with modeling and simulation to guide treatment decisions. One tool derived from a Markov decision model is referenced in the recent 2021 Kidney Disease Improving Global Outcomes (KDIGO) Guidelines for the Management of Glomerular Diseases (GNTools). This tool can help quantify personalized risk and benefit of PAC in patients with risk data derived from the MN cohorts discussed. However, there are important limitations that reflect deficiencies in knowledge available at the time of construction of the tool, and several of these knowledge gaps persist.

First, the risk of VTE events was based on data derived in MN—there were insufficient VTE events in the other GN populations to reliably estimate the risk. The tool should, therefore, *only* be applied in patients with idiopathic MN. Next, the individual bleeding risk is estimated for warfarin (target international normalized ratio 2–3) using the anticoagulation and risk factors in atrial fibrillation score.^6^ This validated tool was selected because the variables required to calculate risk were uniformly available within the source MN cohorts, and the demographics of the anticoagulation and risk factors in atrial fibrillation population were reasonably similar to the MN cohorts. However, studies do suggest that the hypertension, abnormal renal/liver function, stroke, bleeding history or predisposition, labile international normalized ratio [INR], elderly [age ≥65 years], drugs/alcohol concomitantly score better predict bleeding risk in a spectrum of populations.

The drug-specific bleeding risk and efficacy of VTE event prevention remain poorly defined in patients with NS. Data from healthy or general CKD populations are not sufficient to estimate risk and efficacy in NS, where variables, such as hypoalbuminemia, urinary loss of protein/protein-bound drugs, and fluctuation in kidney function, are likely to have important impact on these estimates.

## Aspirin as a Compromise?

Clinicians who are reluctant to expose patients to bleeding risks associated with warfarin may be tempted to use aspirin as an alternative to prevent thrombotic events. Indeed the 2021 KDIGO Guidelines support the use of aspirin as PAC in patients with MN who are at high risk of VTE event but also carry a high bleeding risk and where it is estimated that arterial thrombotic risk is high. Although the rationale for this recommendation acknowledges the surprisingly high cardiovascular risk in patients with GN, data regarding a role for aspirin in prevention of VTE are lacking. In the MN cohorts described, 46% of patients with VTE events were already on an antiplatelet agent (mostly aspirin) at the time of VTE diagnosis, suggesting that this is not a sufficiently effective means of PAC.^3^ This is supported by the finding of evidence of a high rate of *in vitro* resistance to the effects of aspirin on platelets in 83 patients with NS.^7^

The bleeding risk associated with aspirin use in patients with NS is not well defined. Important data derived from a large randomized clinical trial suggest that in patients older than 70 years treated with low-dose aspirin, the risk of major bleeds was significantly higher in patients with eGFR <60 (risk ratio 1.6) and in those with albuminuria (urine albumin-to-creatinine ratio >3 mg/mmol, risk ratio 2.10).^8^

## New Data

Like warfarin, variable protein binding and renal clearance have potential to affect the safety and efficacy of DOACs in patients with NS. Consequently, they are not recommended for PAC in the KDIGO guidelines. Data regarding the bleeding risk associated with DOACs compared with warfarin in patients with CKD are so far reassuring. While prospective studies are still underway, large observational studies suggest that DOACs confer a lower risk of bleeding events even in advanced CKD.^9^

A recent study by Derebail *et al.* is a step forward in improving our understanding of the pharmacokinetics and pharmacodynamics of DOACs in patients with NS.^10^ In this phase 1a study, eight patients with NS (3 MN, four FSGS, one minimal change) and 11 healthy subjects were given a single dose of apixaban, a highly protein-bound DOAC (87%–93% protein bound, 66% bound to albumin). Serial measurements were performed over 24 hours to evaluate drug levels and surrogate markers of drug effect. In the four patients with severe NS (urine protein-to-creatinine ratio >4 g/g and/or albumin <30 g/L), the total apixaban levels were lower in severe NS, and total clearance rate of apixaban was higher. The free apixaban clearance was lower in NS, suggesting that more rapid clearance of total apixaban is driven by a greater clearance of the protein-bound apixaban, potentially due to proteinuria. Patients with NS also had higher maximum free apixaban levels with slower clearance of free apixaban. The anti-Xa activity did not differ compared with control subjects. Both total and free apixaban correlated with anti-FXa concentration. The D-dimer generation at 24 hours was higher in patients with NS.

Taken together, these data suggest that apixaban may be less effective in patients with NS (especially if severe) because they have less total apixaban exposure and increased clearance relative to healthy controls. Higher free apixaban exposure in NS might also subject patients to an increased risk of bleeding. There are several limitations to this study, including the small sample size and no measured impact on clinical outcomes. Furthermore, only a single dose was given; therefore, measures could not be taken at the true steady state. Nonetheless, these data support the need for additional studies to determine the dosing, efficacy, and safety in patients with NS. It should be noted that warfarin pharmacokinetics are also affected by NS,^4^ and this merits further investigation. However, given the convenience of DOACs and favorable safety profile in other populations, better understanding of the applicability of this class of anticoagulants is a priority.

An additional concern regarding the use of DOACs in patients with NS in the past is the potential for interaction with calcineurin inhibitors, which are frequently used to treat NS. Inhibitors of the transporter permeability glycoprotein, such as calcineurin inhibitors, could theoretically increase DOAC levels. Ongoing work in this area supports an interaction between cyclosporine and edoxaban; however, with increasing experience using DOACs in patients on calcineurin inhibitors, clinically important interactions are not clear. Close monitoring of anti-Xa levels is nonetheless recommended if these medications are used together.^11^

## The Path Forward

Further recommendations regarding the risks and benefits of PAC in NS should be driven by controlled trials in patients at high risk of VTE events. The risk:benefit ratio for use of PAC is currently best defined in patients with MN, but the optimal method for safe and effective PAC is not known and is only modeled for warfarin. The KDIGO guidelines, including the use of aspirin for PAC, acknowledge the high risk of cardiovascular events in patients with NS. However as in healthy subjects, the efficacy and safety of primary prevention with empiric use of low-dose aspirin merits closer examination before widespread application.

In the absence of a formal randomized trial, additional insights into the efficacy and safety of DOACs may be gleaned from pharmacokinetic and pharmacodynamic studies in NS populations. Observational or trial data describing treatment of established VTE events in NS populations with DOACs would also offer insights.

To optimize trial design, our knowledge regarding the risk factors for VTE events in patients with NS (disease diagnosis, serum albumin levels, and others) can be applied to target patients most likely to benefit from these medications. Given the convenience of DOAC dosing and monitoring and the safety profile of this class of medication in healthy and CKD cohorts, prioritizing studies of DOACs as PAC in NS is an important next step. Given the rarity of VTE events in patients with NS, prospective studies will require international collaborative effort.
